# Activation of bitter taste receptors (tas2rs) relaxes detrusor smooth muscle and suppresses overactive bladder symptoms

**DOI:** 10.18632/oncotarget.8549

**Published:** 2016-04-02

**Authors:** Kui Zhai, Zhiguang Yang, Xiaofei Zhu, Eric Nyirimigabo, Yue Mi, Yan Wang, Qinghua Liu, Libo Man, Shiliang Wu, Jie Jin, Guangju Ji

**Affiliations:** ^1^ National Laboratory of Biomacromolecules, Institute of Biophysics, Chinese Academy of Sciences, Beijing, China; ^2^ Department of Urology, Beijing Jishuitan Hospital, Beijing, China; ^3^ Department of Urology, National Research Center for Genitourinary Oncology, Peking University First Hospital and Institute of Urology, Beijing, China; ^4^ Department of Gastroenterology, Peking University First Hospital, Beijing, China; ^5^ Institute for Medical Biology, College of Life Sciences, South-Central University for Nationalities, Wuhan, China

**Keywords:** bitter taste receptors, chloroquine, detrusor smooth muscle, human, mouse, overactive bladder, Gerotarget

## Abstract

Bitter taste receptors (TAS2Rs) are traditionally thought to be expressed exclusively on the taste buds of the tongue. However, accumulating evidence has indicated that this receptor family performs non-gustatory functions outside the mouth in addition to taste. Here, we examined the role of TAS2Rs in human and mouse detrusor smooth muscle (DSM). We showed that mRNA for various TAS2R subtypes was expressed in both human and mouse detrusor smooth muscle (DSM) at distinct levels. Chloroquine (CLQ), an agonist for TAS2Rs, concentration-dependently relaxed carbachol- and KCl-induced contractions of human DSM strips. Moreover, 100 μM of CLQ significantly inhibited spontaneous and electrical field stimulation (EFS)-induced contractions of human DSM strips. After a slight contraction, CLQ (1 mM) entirely relaxed carbachol-induced contraction of mouse DSM strips. Furthermore, denatonium and quinine concentration-dependently decreased carbachol-induced contractions of mouse DSM strips. Finally, we demonstrated that CLQ treatment significantly suppressed the overactive bladder (OAB) symptoms of mice with partial bladder outlet obstruction (PBOO). In conclusion, we for the first time provide evidence of the existence of TAS2Rs in the urinary DSM and demonstrate that TAS2Rs may represent a potential target for OAB. These findings open a new approach to develop drugs for OAB in the future.

## INTRODUCTION

The urinary bladder is the organ that collects urine excreted from kidneys before disposal by urination. Bladder dysfunction, such as overactive bladder (OAB), has serious effects on quality of life [1]. OAB syndrome is a common condition characterized by the presence of urgency with or without incontinence, frequency, and nocturia. The etiology of OAB syndrome is very complicated and includes increased afferent activity, decreased inhibitory control, and increased sensitivity of the detrusor muscle to efferent stimulation [2]. It has been reported that the overall prevalence of OAB syndrome was 11.8%; rates were similar between men and women [3]. The prevalence of OAB syndrome increased with age in a linear fashion [3-5]. As a result, in patients over the age of 65 years the prevalence of OAB syndrome can increase to 30.9% [4]. Anticholinergic drugs remain the first-line pharmacologic treatment for OAB syndrome [6] despite producing undesirable side effects such as dry mouth, constipation, and blurred vision [7]. It is estimated that by 2018, more than 500 million people worldwide will be affected by OAB [3, 8]. Thus, it is of great urgency to identify novel targets for this disorder.

Bitter taste receptors (TAS2Rs) belong to the superfamily of G-protein-coupled receptors (GPCRs) [9]. GPCRs, which convert extracellular stimuli into intracellular signals through the activation of heterotrimeric G-proteins, are involved in many diseases and are also the target of approximately 40% of all modern medicinal drugs [10]. However, these targeted GPCRs are only a small part of this receptor superfamily and over 30% GPCRs have no known endogenous ligand, indicating that many potential targets remain to be discovered [11]. As TAS2Rs are traditionally thought to be expressed exclusively on the taste buds of the tongue, they have been generally neglected as drug targets. However, accumulating evidence has indicated that this receptor family performs non-gustatory functions outside the mouth in addition to taste. We and others have shown that TAS2Rs are expressed in both human and mouse airway smooth muscle and mediate the tone of airway smooth muscle [12-15]. Besides, it has been reported that this receptor family plays a critical role in the heart [16], thyroid [17], and gastrointestinal muscle [18]. However, their role in the urinary bladder has never been determined.

In this study, we examined the expression and function of TAS2Rs in human and mouse urinary detrusor smooth muscle (DSM). We further determined their role in the urinary bladder of mice with partial bladder outlet obstruction (PBOO).

## RESULTS

### Expression profile of *Tas2rs* in human DSM

**Figure 1:**
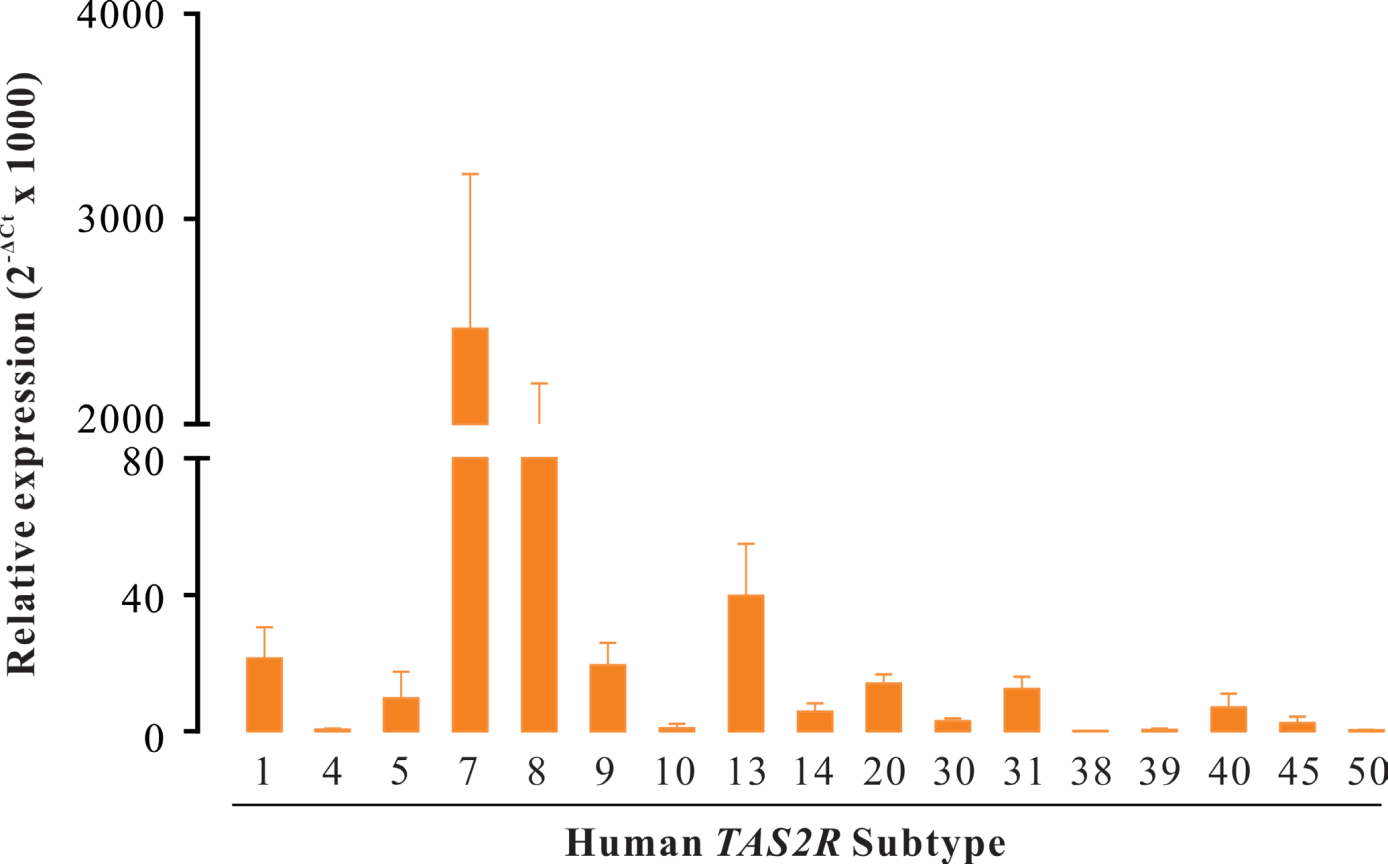
The genes of *TAS2R* are expressed in human DSM RT-qPCR screen of *TAS2R* genes in human DSM. Data were presented as relative expression of *TAS2R* genes to the reference gene *GAPDH* (mean ± SEM of 5 independent DSM samples). Of all 25 human *TAS2R* gens, 17 *TAS2Rs* were expressed in human DSM with distinct levels, whereas 8 *TAS2R*s were not detected.

Using quantitative reverse transcription PCR (RT-qPCR), we screened the expression profile of all 25 *TAS2R* genes in human DSM. As shown in Figure 1, we found that *TAS2R7* and *TAS2R8* were the most abundantly expressed genes, with levels similar to the reference gene glyceraldehyde 3-phosphate dehydrogenase (*GAPDH*). The expression levels of *TAS2R1*, *TAS2R5*, *TAS2R9*, *TAS2R13*, *TAS2R20*, and *TAS2R31* were two orders of magnitude lower than that of *GAPDH* with the following rank order: *TAS2R13* > *TAS2R1*≈*TAS2R9* > *TAS2R5*≈*TAS2R20*≈*TAS2R31*. *TAS2R4*, *TAS2R10*, *TAS2R14*, *TAS2R30*, *TAS2R38*, *TAS2R39*, *TAS2R40*, *TAS2R45*, and *TAS2R50* were very slightly detected. Eight *TAS2R* genes (*TAS2R3*, *TAS2R16*, *TAS2R19*, *TAS2R41*, *TAS2R42*, *TAS2R43*, *TAS2R46*, and *TAS2R60*) were not expressed.

### Chloroquine (CLQ) relaxes human DSM strips

**Figure 2:**
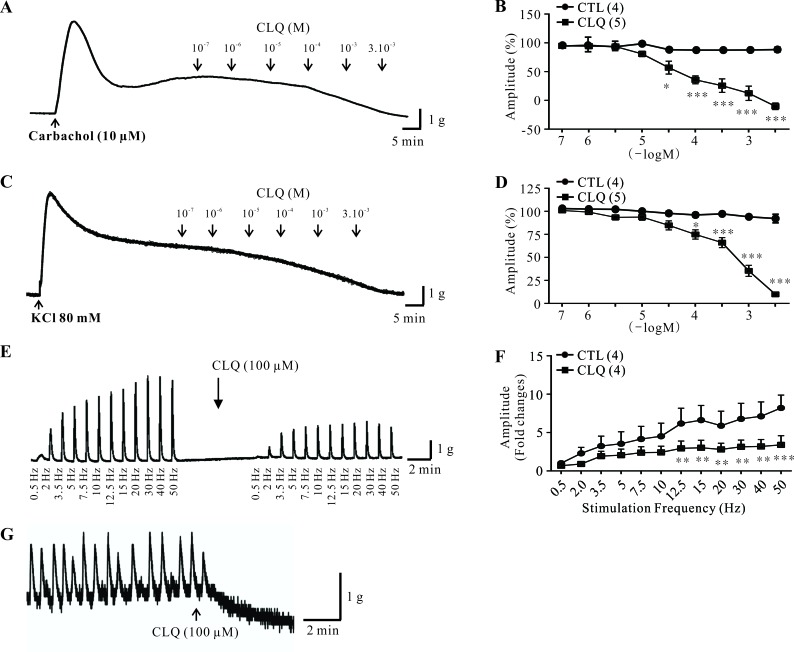
Effect of chloroquine on stimulus-induced and spontaneous contractions of human DSM strips **A.**, **B.** Original trace and summary data showing the effects of vehicle (control, *n* = 4 strips) or chloroquine (CLQ: 100 nM to 3 mM; *n* = 5 strips) on carbachol-induced contractions. **C.**, **D.** Original trace and summary data showing the effects of vehicle (control, *n* = 4 strips) or CLQ (100 nM to 3 mM; *n* = 5 strips) on KCl-induced contractions. **E.**, **F.** Original trace and summary data showing the effects of CLQ (100 μM) on EFS-induced contractions (*n*= 4 strips). **G.** Original trace of spontaneous contractions in the absence and presence of 100 μM CLQ. Data are mean ± SEM of n independent DSM strips. *p < 0.05, **p < 0.01, and***p < 0.001 as indicated.

**Figure 3:**
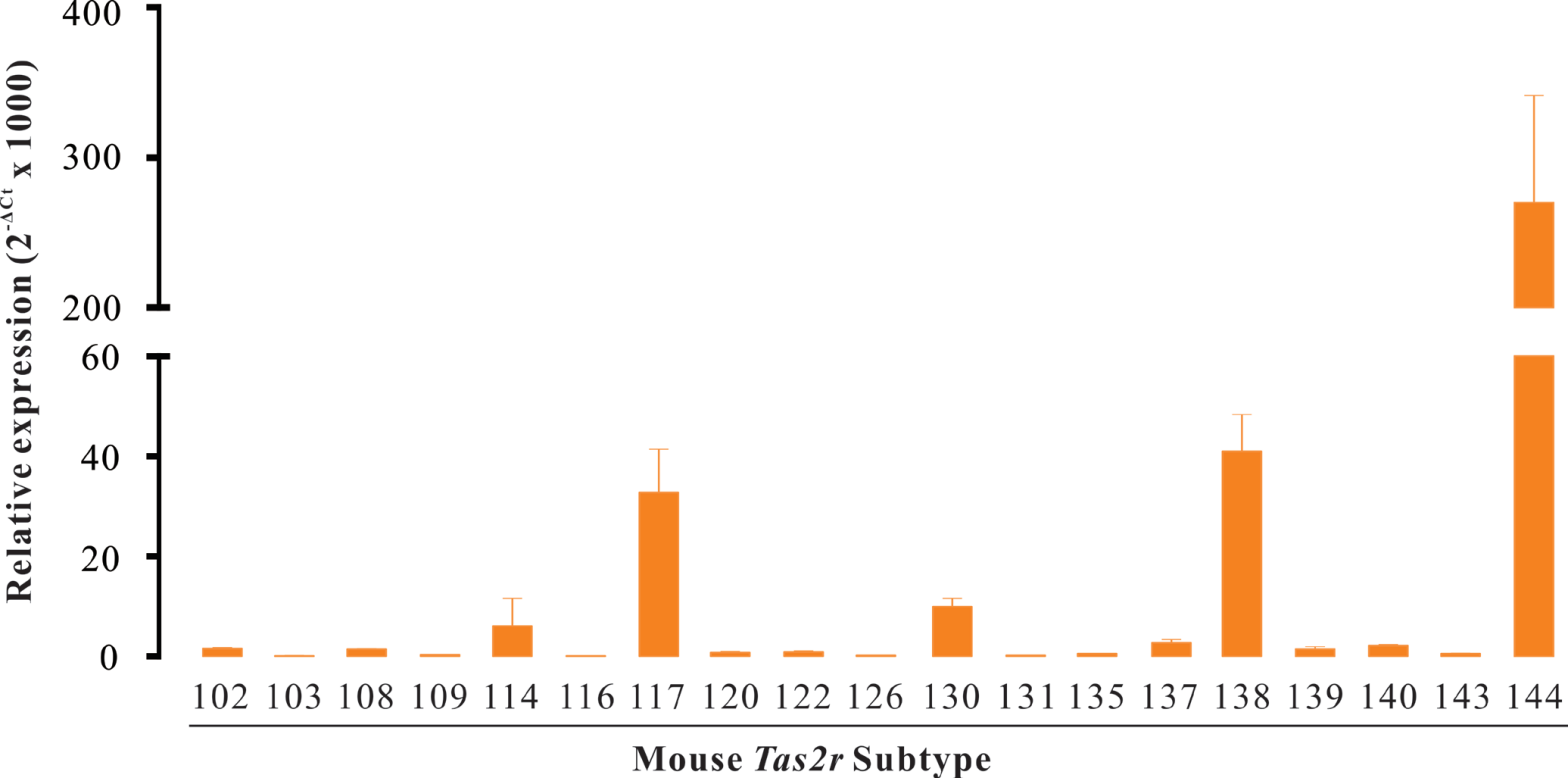
The genes of *Tas2r* are expressed in mouse DSM RT-qPCR screen of *Tas2r* genes in mouse DSM. Data were presented as relative expression of *Tas2r* genes to the reference gene *Gapdh* (mean ± SEM of 3 independent DSM samples). Of all 35 mouse *Tas2r* genes, 19 *Tas2rs* were expressed in mouse DSM with distinct levels, whereas 16 *Tas2r*s were not detected.

Next, we determined the function of TAS2Rs in human DSM with chloroquine (CLQ). CLQ is an agonist of TAS2Rs and has been shown to activate several TAS2Rs, including TAS2R3, TAS2R7, TAS2R10, and TAS2R39 [19-21]. We showed that CLQ did not exhibit any effects on the base tone of human DSM strips. We thus tested the effect of CLQ on carbachol- and KCl-induced contractions of human DSM strips according to previous studies [22-24]. We showed that cumulative concentrations of CLQ (100 nM to 3 mM) induced significant decreases of carbachol- and KCl-induced contractions in a concentration-dependent manner (Figure 2A-2D). In contrast, the vehicle had no significant effects on these contractions (Figure 2B and 2D). Moreover, we investigated the effects of CLQ on the nerve-evoked contractions induced by a wide range of electrical field stimulation (EFS) frequencies as described from Petkov's work [25]. We first applied increasing EFS frequencies (0.5-50 Hz) as a control protocol, followed by the addition of 100 μM CLQ (Figure 3E). Five minutes after the addition, a second EFS protocol was applied. As shown in Figure 3F, CLQ (100 μM) significantly decreased the amplitudes of EFS-induced contractions within a wide range (0.5 Hz, 2 Hz, 3.5 Hz, 5 Hz, 7.5 Hz, 10 Hz, 12.5 Hz, 15 Hz, 20 Hz, 30 Hz, 40 Hz, and 50 Hz) by 21 ± 12%, 52 ± 15%, 26 ± 14%, 32 ± 10%, 31 ± 10%, 40 ± 5%, 50 ± 8%, 55 8%, 46 ± 10%, 48 ± 13%, and 57 ± 19%, respectively. Of all the strips (*n* = 26), only three exhibited spontaneous phasic contractions. We found that CLQ (100 μM) completely inhibited these spontaneous phasic contractions (Figure 2G). Taken together, these results suggested that TAS2R activation relaxes human DSM.

### Expression profile of *Tas2rs* in mouse DSM

We further explored the expression profile of *Tas2rs* in mouse DSM with RT-qPCR. Of 35 mouse *Tas2r* genes, *Tas2r114*, *Tas2r117*, *Tas2r130*, *Tas2r138*, and *Tas2r144* were significantly expressed. Among them, only *Tas2r114* was expressed at a similar level to *Gapdh*. Fourteen *Tas2r* genes were very lowly expressed, whereas 16 *Tas2r* genes were not detected (Figure 3).

### Effects of bitter tastants on carbachol-induced contractions in mouse DSM strips

**Figure 4:**
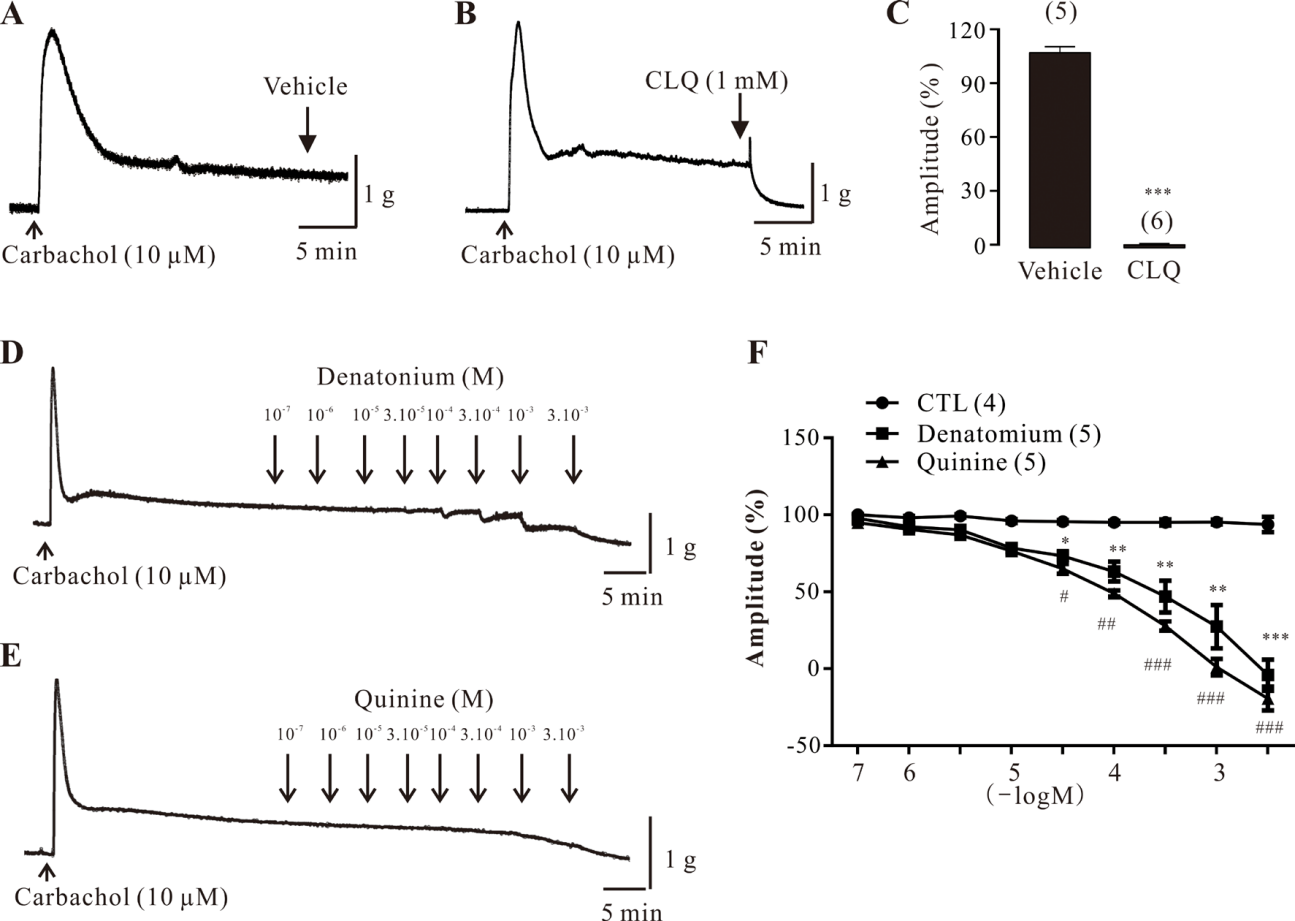
Effect of bitter tastants on carbachol-induced contractions of mouse DSM strips **A.-C.** Original traces and summary data showing the effects of vehicle (control, *n* = 5 strips) or chloroquine (CLQ: 1 mM; *n* = 6 strips) on carbachol-induced contractions in mouse DSM strips. **D.** Original trace showing the effects of denatonium (100 nM to 3 mM) on carbachol-induced contractions. **E.** Original trace showing the effects of quinine (100 nM to 3 mM) on carbachol-induced contractions. **F.** Summary data showing the effects of vehicle, denatonium (*n* = 5 strips), and quinine (*n* = 5 strips) on carbachol-induced contractions. Data are mean ± SEM of n independent mouse DSM strips. *p < 0.05, **p < 0.01, and ***p < 0.001 were control *vs*. CLQ or denatomium; #*p* < 0.05, ##*p* < 0.01, and ###*p* < 0.001 were control *vs*. quinine, respectively.

To determine the role of TAS2Rs in mouse DSM, three different bitter tastants, CLQ, denatonium, and quinine, were used. As shown in Figure 4A-4C, we found that CLQ (1 mM) mediated a two-phasic response: it first produced a slight contraction and then completely relaxed the carbachol-induced contractions. Moreover, both denatonium and quinine concentration-dependently decreased carbachol-induced contractions of mouse DSM strips (Figure 4D-4F). The vehicle had no significant effects on these contractions (Figure 4F).

### Chloroquine treatment attenuates bladder morphological alteration of PBOO mice

**Figure 5:**
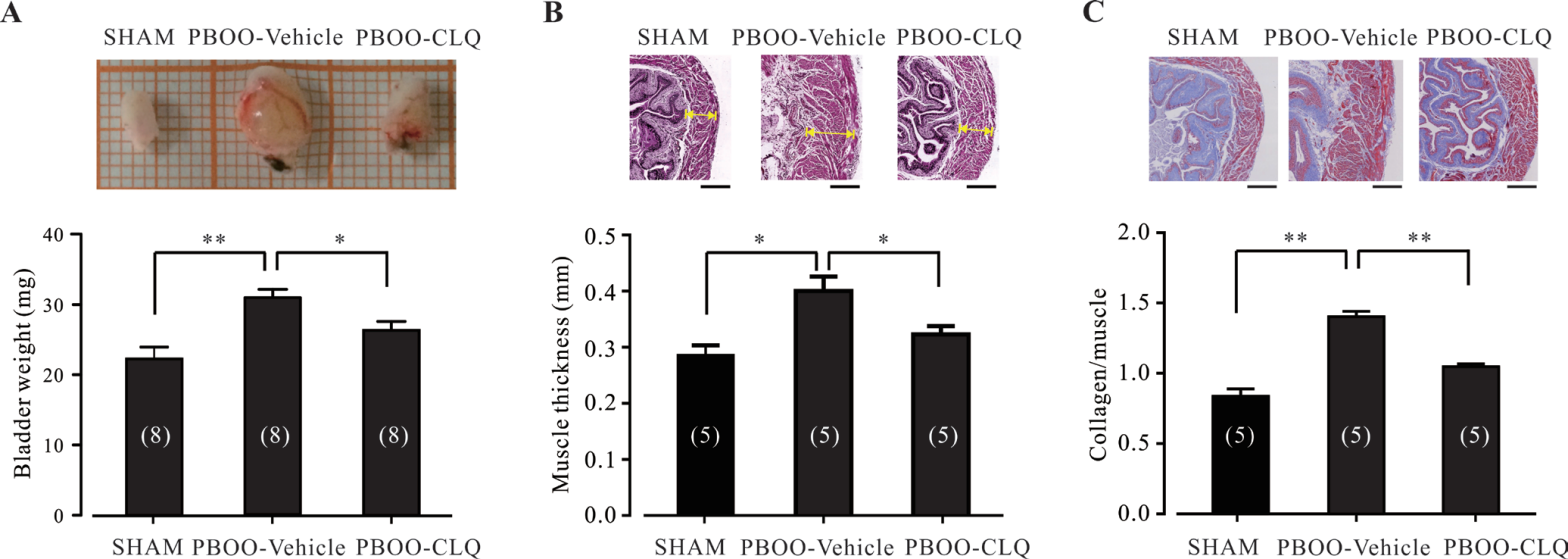
Chloroquine treatment significantly attenuates the bladder morphological changes of PBOO mice **A.** Bladder weights of the sham (*N* = 8), PBOO-Vehicle (*N* = 8), and PBOO-CLQ (*N* = 8) mice are shown. After anesthesia, the whole bladder was removed and imaged. The fat and connective tissues were cleaned under cold PBS. Then, the bladder was cut from the neck to the fundus along one side. After being quickly dried by drinking paper, the bladder was weighed. **B.** The representative HE stains of bladder specimens from the sham, PBOO-Vehicle, and PBOO-CLQ mice are shown. Summary data showing the average muscle thickness of bladders from the sham (*N* = 5), PBOO-Vehicle (*N* = 5), and PBOO-CLQ (*N* = 5) mice. **C.** The representative Masson's trichrome staining of bladder from the sham, PBOO-Vehicle, and PBOO-CLQ mice were shown. Summary data showing the mean ratio of collagen to muscle from the sham (*N* = 5), PBOO-Vehicle (*N* = 5), and PBOO-CLQ (*N* = 5) mice. Scale bars = 250 μm. *p < 0.05 and **p < 0.01 as indicated.

It has been well accepted that detrusor over-activity is one of the major causes of OAB syndrome [2]. Thus, relaxation of the urinary DSM has been recognized as an effective approach to treat OAB. Our *in vitro* results demonstrated that TAS2Rs activation can relax both human and mouse urinary DSM (Figures 2 and 4). We therefore hypothesized that TAS2Rs activation would be effective for the treatment of OAB. To test this hypothesis, we induced a mouse model of OAB through partial obstruction of the urinary bladder outlet. The mean bodyweights of the sham, PBOO-vehicle, and PBOO-CLQ mice were not significantly different (data not shown). As shown in Figure 5A, the bladder weights of PBOO-vehicle mice (*N* = 8; 0.031 ± 0.002 g) were significantly increased than that of sham mice (*N* = 8; 0.022 ± 0.002 g); CLQ treatment markedly suppressed this increase (PBOO-CLQ mice: *N* = 8; 0.026 ± 0.001 g). Moreover, hematoxylin-eosin (HE) staining indicated that the bladder muscle thicknesses of PBOO-vehicle mice (*N* = 5; 0.40 ± 0.03 mm) were markedly thicker than that of sham mice (*N* = 5; 0.28 ± 0.02 mm); while CLQ treatment largely prevented PBOO-mediated muscle hypertrophy (PBOO-CLQ mice: *N* = 5; 0.32 ± 0.01 mm) (Figure 5B). In all groups, muscle fibers (stained red) and collagen fibers (stained blue) can be clearly observed in the bladder wall (Figure 5C). Consistent with HE results, masson's trichrome staining also indicated that the bladder walls were thicker in PBOO-vehicle and PBOO-CLQ mice in comparison with sham mice. Additionally, there was a transmural increase in the amount of collagen in both PBOO-vehicle and PBOO-CLQ mice. The collagen/muscle ratio in the muscle layer of bladder wall was significantly increased in PBOO-vehicle mice (*N* = 5; 1.4 ± 0.04) compared to sham mice (*N* = 5; 0.84 ± 0.05); this increase was markedly suppressed by CLQ treatment (PBOO-CLQ mice: *N* = 5; 1.05 ± 0.05).

### Chloroquine treatment improves bladder function of PBOO mice

**Figure 6:**
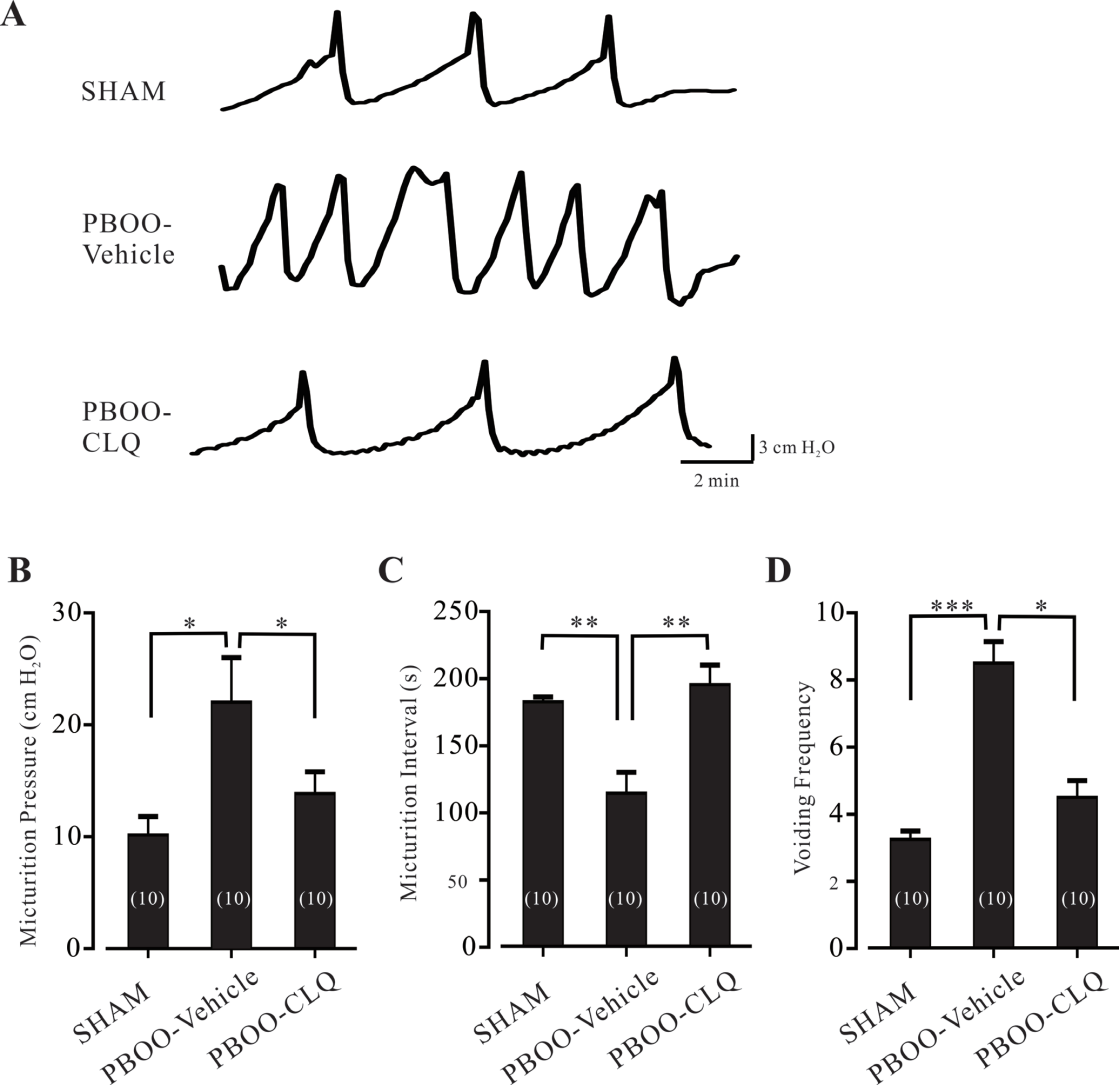
Chloroquine treatment improves the bladder function of PBOO mice **A.** The representative traces of the cystometrogram recorded in the sham, PBOO-Vehicle, and PBOO-CLQ mice. **B.-D.** Summary data of micturition pressures, micturition intervals, and voiding frequency of the sham (*N* = 10), PBOO-Vehicle (*N* = 10), and PBOO-CLQ (*N* = 10) mice are shown. Data are mean ± SEM. *p < 0.05, **p < 0.01, and ***p < 0.001 as indicated.

We also explored the bladder function of PBOO mice after CLQ treatment. As shown in Figure 6A, compared to sham mice, PBOO-Vehicle mice exhibited aberrant urodynamic manifestations; while PBOO-CLQ mice had normal urodynamic features. In detail, maximum micturition pressure of PBOO-Vehicle mice was significantly higher than that of sham mice; CLQ treatment suppressed this increase (Figure 6B). PBOO significantly shortened the micturition interval; however, these values were significantly prolonged and increased by CLQ treatment (Figure 6C). The micturition frequency of PBOO-Vehicle mice was largely increased compared to sham mice; this value was markedly decreased by CLQ treatment (Figure 6D).

## DISCUSSION

In this study, we demonstrated for the first time that TAS2Rs exist in human and mouse DSM based on both molecular and pharmacological studies. With RT-qPCR, we determined the expression profile of *TAS2Rs* in human and mouse DSM (Figures 1 and 3). We showed that *TAS2R7* and *TAS2R8* were the main subtypes expressed in human DSM (Figure 1). The murine homolog of *TAS2R7*, *Tas2r130*, was significantly detected in mouse DSM (Figure 3). Likewise, *Tas2r144* was the major subtype expressed in mouse DSM (Figure 3). Its human homolog, *TAS2R40*, was also observed in human DSM (Figure 1). However, a large number of *TAS2R* subtypes expressed in human DSM were not detected in mouse DSM. It is perhaps due to the fact that most of them are human specific *TAS2Rs* including *TAS2R5*, *TAS2R8*, *TAS2R9*, *TAS2R30*, *TAS2R31*, and *TAS2R45*. With a classical pharmacological method, we showed that TAS2Rs activation induces a strong relaxation of both human and mouse DSM strips. Of interest, a transient contraction before the relaxation was observed in mouse DSM strips (Figure 4). However, this phenomenon did not occurred in human DSM. These results suggested that TAS2Rs and their downstream signaling pathways might be varied among species. Like CLQ, the other two bitter tastants, quinine and denatonium, largely relaxed the carbachol-induced contractions of mouse DSM strips in a concentration dependent manner (Figure 4D-4F), confirming the existence of TAS2Rs in mouse DSM. We also found that CLQ had no obvious effects on the base tone but suppressed stimulus-induced contractions of DSM, implying that the underlying signaling pathways might be different between the resting and pre-contracted conditions. It has been shown that TAS2Rs activation mediates two opposing signaling pathways in the airway smooth muscle [14].

In the airway epithelium, TAS2Rs expressed on the ciliated epithelial cells [26] and solitary chemosensory cells [27, 28], where they promote ciliary beat frequency and sense chemical irritation, respectively. Here, we did not focus the role of TAS2Rs in the urothelium of urinary bladder. It is hard to obtain the human bladder tissues with normal urothelium in our experimental conditions. In most cases, only the DSM sections were delivered to the lab from the hospital. In order to keep consistent with findings in the human urinary DSM, we only investigated the role of TAS2Rs in mouse DSM. However, it will be very interesting to test the role of TAS2Rs in the urothelium of urinary bladder.

Another primary finding of this study is that CLQ treatment suppresses OAB symptoms. We showed that once daily 30 mg/kg of CLQ for 6 weeks can attenuate PBOO-induced DSM hypertrophy and collagen deposition (Figure 5) and improve bladder functions of PBOO mice (Figure 6). The dosage of CLQ used in this study was based on previous observations. Sexton et al. showed that 25 and 50 mg/kg of CLQ are effective for the treatment of human malaria [29]. In a murine malaria model, a single dose of 50 mg/kg of CLQ can protect mice from experimental malaria [30]. It has been reported that once daily 30 mg/kg of CLQ has no significant effects on the behavior and appearance of the mice [31]. Moore et al. found that the maximum plasma concentration of CLQ can reach to 1 708 μg/liter (about 6 μM) after the treatment of a single dose of CLQ (50 mg/kg) in the mice [32]. CLQ concentration can be accumulated more than 10 times after the successive treatment of CLQ for 20 days in human [33]. As the pharmacokinetics of CLQ is similar between human and mouse [32], we calculated that the plasma concentration of CLQ should be maintained from 10 to 100 μM during CLQ treatment. After PBOO surgery, the bladder underwent structural and functional remodeling to adapt the excessive mechanical stress [34]. One of the major functional alterations caused by PBOO is detrusor over-activity, which is characterized by increased responses to stimuli and micturition dysfunction. The super-sensitivity of the detrusor to acetylcholine (Ach) has been reported in the animal model [35] as well as in OAB patients [36]. Thus, CLQ treatment suppresses detrusor over-activity and leads to the improvement of the bladder function of PBOO mice. Recently, Long et al. showed that CLQ can prevent the hypertrophy of right ventricular through the inhibition of autophagy pathways [37], raising the possibility that there are the other mechanisms responsible for the effects of CLQ treatment.

In this study, the OAB model was generated with female mice because of the ease of manipulation. It is known that animal models always have several limitations, including the fact the prevalence of bladder obstruction is higher in males than in females. Besides the obstruction of bladder outlet, there are numerous causes of OAB in human. Thus, the effects of CLQ needs to be further investigated with the other OAB models.

There is an unmet need for additional therapeutic options in the treatment of OAB. Here, we provide evidence of the existence of TAS2Rs in both human and mouse urinary DSM and further demonstrate that CLQ can improve OAB symptoms of PBOO mice. Indeed, many known synthetic agents and thousands of natural bitter tastants and their metabolites can activate TAS2Rs and have favorable therapeutic profiles. Therefore, these findings open a new approach to develop drugs for OAB in the future.

## MATERIALS AND METHODS

This study was reviewed and approved by the local Institutional Review Board of Peking University First Hospital and the Institute of Biophysics Committee. For experiments involving human subjects, the research was conducted according to the principles expressed in the Declaration of Helsinki. Animal protocols were performed according to the Guide for the Care and Use of Laboratory Animals published by the US National Institutes of Health (NIH Publication No. 85-23, revised 1996).

### Obtainment of human urinary bladder tissues

Human urinary bladder tissues were obtained from 26 patients with bladder cancer (18 men and 8 women, mean age: 66 ± 7 yr) who had undergone radical cystectomy in Peking University First Hospital. The patients with urinary retention, obvious lower urinary tract symptoms, or with medical conditions that could affect bladder function were excluded. In addition, the patients whose DSM layers were severely affected by cancer were also excluded. Bladder tissues were removed and confirmed to be cancer-free by an experienced urological pathologist. After the cleaning of fat tissue and peeling of bladder serosa and mucosa, human DSM samples were collected.

Adult C57BL/6J mice (8-week old, 18-22 g) were purchased from Vital River Laboratories (Beijing, China), housed with free access to food and water and maintained on a 12 hour light/dark cycle. Mice were anesthetized by 5% chloral hydrate and the whole bladder was removed, placed into cold Tyrode solution composed of (mM): 137 NaCl, 5.4 KCl, 1.8 CaCl_2_, 1.0 MgCl_2_, 10 glucose, 10 HEPES, (pH 7.4). After the cleaning of the fat tissues, the bladder was cut into two longitudinal pieces by using a fine dissecting scissors along the axis from the neck to the fundus. Then, the urothelium was carefully removed. The DSM tissues were collected for organ baths or RT-qPCR experiments.

### RT-qPCR

RNA and cDNA were prepared as previously reported [38]. In brief, total RNA was prepared from bladder DSM using the Trizol RNA purification system (Invitrogen, Carlsbad, CA, USA). The cDNA was generated from mRNA (2 μg) using the M-MLV reverse transcriptase (Promega Corp., Madison, WI, USA). RT-qPCR was performed on a Corbett Rotor-Gene 6600 QPCR system machine (Corbett Life Science) using TransScript™ Green RT-qPCR SuperMix (TRANSGENE BIOTECH, Beijing, China) according to the manufacturer's instructions. GAPDH was used as a reference gene. Detailed information regarding the primer pairs used in this study was shown in Table 1. All the PCR products were run on the gel or sequenced to test the specificity of each pair of primer. The 2^−ΔCt^ method was used to analyze the relative expression levels of TAS2Rs.

**Table 1:** Sequence information of RT-qPCR primers

Gene symbol	Accession number	Forward sequence	Reverse sequence	Size (bp)
TAS2R1	NM_019599.2	tgccattgcttatcttccttttt	ggtgtgcctccccagaga	62
TAS2R3	NM_016943.2	caaaaaccaagatggctaagatga	tgagtggccagcaggataaaa	65
TAS2R4	NM_016944.1	tttcctgaacttgtgactacgagaa	taaagacaagatgccctcactgata	66
TAS2R5	NM_018980.2	cagcattcggtatccctttga	tcctgaattgagctgaaatgca	61
TAS2R7	NM_023919.2	aacgctgctccccttttgt	cgcagggagaggatcaagag	60
TAS2R8	NM_023918.1	aaaactctatgctaccggcagtaga	agtcatagttttaatggctctcacatg	70
TAS2R9	NM_023917.2	tgcatgctacagggttcagaga	tgcctttatggccctcatgt	59
TAS2R10	NM_023921.1	catttccctttggagacacaac	atgagcttctgtgttggagtc	76
TAS2R13	NM_023920.2	aggagcagaaaaaggagaagg	gtgaagatactcggcaggg	147
TAS2R14	NM_023922.1	cctcactgctttggcaatctc	acacacaccagcttccgaatatt	65
TAS2R16	NM_016945.2	cattggttattcctttcatcctgtt	cttggtcagtgatgccatgaga	65
TAS2R19	NM_176888.1	cgaaccatttcagcatgtgg	ccccaacagtatcaccagaac	134
TAS2R20	NM_176889.2	agatggagtcttgccctgttgt	ttgtggtgagccaagattgtg	62
TAS2R30	NM_001097643.1	atttcagcagctggcttgcta	aaattggcaatcctgagcaaa	61
TAS2R31	NM_176885.2	cagcaccaaggtccacataaaa	gtaaacggcacataacaagaggaa	67
TAS2R38	NM_176817.4	ctctgtgcccctactgattctgt	cattatcccaacacaaaccatcac	64
TAS2R39	NM_176881.2	ccctgccagccactcaat	ccgcttccaggctcttctc	64
TAS2R40	NM_176882.1	tgccggccactcagtacaa	accgcttccaggctcttctc	62
TAS2R41	NM_176883.2	cggccgacagttcttcca	aaaaccagaaggtggctgagttc	59
TAS2R42	NM_181429.1	actggtaaactgctctgaagg	atgtgaagcaagtcccactag	139
TAS2R43	NM_176884.2	gcaccaaggtccacataaaagc	aagtaaatggcacataacaagaggaa	67
TAS2R45	NM_176886.1	cctttgctgaccaaattgtcact	taataataacacccagagcaaaccaa	68
TAS2R46	NM_176887.2	gctattgcattcagctatccttca	agcttcttgtttccccaaatca	62
TAS2R50	NM_176890.2	gttgtcatggttagcaaggc	gagttgagagtttcaggtcttttac	148
TAS2R60	NM_177437.1	caatgcccactgctgtctttt	tgtgtcttcccagagatgtgatg	63
hGAPDH	NM_002046.5	gccacatcgctcagacacc	cccaatacgaccaaatccgt	64
Tas2r102	NM_199153.2	ggaagcttggtgttcttgcttgg	agatcagctcggtccacattgc	127
Tas2r103	NM_053211.1	attagcactgggtttacactcacc	ccacagggagaagatgagcagaag	75
Tas2r104	NM_207011.1	agcttcctttccgctagctgtg	tggatcagccaggatgtgttgc	75
Tas2r105	NM_020501.1	ttccttctcatcggcttagca	gtcaggtgattcacagtcatcc	152
Tas2r106	NM_207016.1	tgcctctgatgcccacattatag	ggctggtggcaaaccatatacttg	80
Tas2r107	NM_199154.1	tccctgcggtcactcaatcatc	cagtgccttcaaagaggcttgc	70
Tas2r108	NM_020502.1	agtgtttctcctgttgaaacggac	tggtgagggctgaaatcagaag	83
Tas2r109	NM_207017.1	gtcaaattcaggtgttaggaagtcac	cacagggagaagatgagcagga	94
Tas2r110	NM_199155.2	tggatagtgaataaccatttcagcg	ctccactttaggtaaagaaacaaagagt	110
Tas2r113	NM_207018.1	tccgcactgctctggcaattag	tgaacagacacccaccaatctagg	73
Tas2r114	NM_207019.1	tgctgagcacaatggaaggtgtc	tgttccctacaatgcccagcac	72
Tas2r115	NM_207020.1	ctttggtgtatccttgatagctttcc	gtactgcatcttccttacatgtttcat	73
Tas2r116	NM_053212.1	cttttgctgtgtcactggtca	tctgatgtgggccttagtgct	119
Tas2r117	NM_207021.1	cttttcgttgtattttgtgaggttgt	ctgtctcagcttcatgtctcctaca	90
Tas2r118	NM_207022.1	aagttgcacaacggttgcagtg	tctccaccggtgacagtctttg	68
Tas2r119	NM_020503.2	ctcaaggaacccaagactcagtg	acaggcttctgagcaggatgtc	82
Tas2r120	NM_207023.1	atggcaaaggatgtcaagatcag	atgacctgctgggtagaagga	182
Tas2r121	NM_207024.1	ctggtcttattggagatgattgtgtt	ggagaagattaacaggatgaaggaga	81
Tas2r122	NM_001039128.1	tcttctctttatggagccaccttag	gtgcttctgtgcttatgtctttgg	75
Tas2r123	NM_207025.1	cattaaagccttgcaaactgtgttc	ggaaaagtaagtatatggcatacagca	62
Tas2r124	NM_207026.1	agtctctggcttgctacagctc	agcttcccagaagcatgtggac	127
Tas2r125	NM_207027.1	atcttctccctgtggagacacctg	tggtgtcttcggagcctttagc	64
Tas2r126	NM_207028.1	gcagtgtgtgggattggtcaac	tcccggagtactcaaccagatg	62
Tas2r129	NM_207029.1	ttgcagatgcccacatcagagtc	tggcacagagtaggacataggtg	60
Tas2r130	NM_199156.1	tccttcctggccctgtttg	tgaatggcttgaaggatagattagag	132
Tas2r131	NM_207030.1	atcaacatggcttgccacctg	agcacacctctcaatctccactta	105
Tas2r134	NM_199158.1	gcctgggaagtggtaacctaca	gtgttgcttagtatcagaatggtgga	63
Tas2r135	NM_199159.1	tcagttctgccagcaacacacc	tgaatcaccacctgccacatcc	64
Tas2r136	NM_181276.1	tctggaggaaccaatccacctg	tgctctcacctgaaccattgcc	133
Tas2r137	NM_001025385.1	ctggctcaaatggagagcttcta	ggtactgacacaggataagagcagtg	76
Tas2r138	NM_001001451.1	tgctattcagctcgcctgcttc	tggcttggtagttgtggctcag	62
Tas2r139	NM_181275.1	tgacaatgttcgtcgcaacagc	tcatgttcagggtgtgtctcctg	66
Tas2r140	NM_021562.1	catctgaagaacatgcaacacaatg	gcagggccttaatatgggct	73
Tas2r143	NM_001001452.1	ttcccaggctgctggttgtatc	agttcccggtggctgaaatgac	69
Tas2r144	NM_001001453.1	tggtttgctgcttggctcaatg	tcagaaggaacagagggtgagc	73
mGapdh	NM_008084.2	aaggtcggtgtgaacggatttg	tcctggaagatggtgatgggct	224

### Isometric DSM tension recordings

Organ bath experiments were performed as previously reported [39, 40]. In brief, the mucosa-free DSM strips from human or mouse were dissected into strips 5-8 mm long and 2-3 mm wide. Strips were tied up and mounted in the standard organ bath chambers (BL-420F acquisition system, Chengdu TME Technology Co, Ltd, Sichuan, China) filled with KREBs solution maintained at 37°C and aerated with 95% O_2_ and 5% CO_2_. DSM strips were passively stretched and equilibrated for 1.5 hour. After equilibration, KCl (80 mM) or carbachol (10 μM) were applied to pre-contract the DSM strips. CLQ, denatonium, and quinine were prepared as 1, 000x in MilliQ water just before use. The equal volume of MilliQ water was used as positive controls. When the contraction responses were stable, cumulative CLQ (100 nM to 3 mM) or the positive controls was added to the chambers. To analyze the effects of CLQ, denatonium, and quinine, the stable contractions induced by KCl (80 mM) or carbachol (10 μM) was taken as 100% and the responses to subsequent bitter tastants or control applications were normalized. For EFS experiments, DSM contractions were generated by applying increasing EFS frequencies (0.5, 2, 3.5, 5, 7.5, 10, 12.5, 15, 20, 30, 40, 50 Hz). EFS pulses were generated using the BL-420F acquisition system (Chengdu TME Technology Co, Ltd) and had the following parameters: pulse amplitude was 20 V, pulse width was 0.75 ms, stimulus duration was 3 s, and polarity was reversed for alternating pulses according to a previous study [25]. Five minutes after the addition of 100 μM CLQ, a second EFS stimulation with same frequencies was applied. To analyze the effects of CLQ on the EFS generated contractions, the contraction amplitude at 0.5 Hz of the first EFS stimulation was taken as one and the other contraction amplitudes were normalized to it and shown as fold changes.

### Partial bladder outlet obstruction

The OAB mouse model was induced with female C57BL/6J mice (8-week old, 18-22g) as previously described [41]. In brief, mice were anesthetized with 5% chloral hydrate, then abdomen was opened by a lower midline incision. The bladder and urethra were carefully separated from the surrounding tissues. After placing a 0.5-mm metal rod alongside the proximal urethra, a 4-0 nylon ligature was tied both the rod and the urethra to make an infravesical obstruction. The rod was subsequently removed, before closing the abdomen. Sham-operated animals served as controls. Two weeks after surgery, the PBOO mice were divided into two subgroups as follows: one group received once 30 mg/kg of CLQ for 6 weeks *via* oral gavage (as PBOO-CLQ mice), and the other group received normal saline (as PBOO-Vehicle mice). The sham-operated mice received normal saline only.

### Histological examination

The urinary bladders were fixed with 4% paraformaldehyde, embedded in paraffin and sectioned (5 μm) onto glass slides. HE staining and Masson's trichrome staining were carried out by using standard protocols. Images were acquired on a Leica SCN400 Scanner microscope (Leica). To determine the bladder muscle thickness, images of HE staining were analyzed by Image J. Nine non-overlapping fields per mouse bladder were measured and the mean of the bladder muscle thickness was calculated. To measure the ratio of collagen/muscle in the bladder muscle layer, images of masson's trichrome staining were analyzed by using Image Pro Plus software. Through color recognition, this system automatically measures the area of each color. Nine fields per mouse bladder were manually chose and analyzed. This analysis was carried out by a single person, who was completely blinded to the experimental data and all other variables.

### Cystometry

As reported previously [42], the mice underwent a surgical procedure for catheter (PE-10) insertion. The mice were anesthetized with 5% chloral hydrate. The abdomen was opened and a PE-10 catheter was inserted into the bladder and fixed with a surgeon's knot. After closure the muscle, the catheter was tunneled subcutaneously and an orifice made at the back of the animal. Three days after surgery, conscious mice were placed in cages without any restraints. The PE-10 tube was connected to a pressure transducer (LABORIE, Canada) and an infusion pump (B. Braun Medical, Inc., Germany) through a 3-way tap. Saline solution (37°C) was infused into the bladder at a rate of 1.2 ml/h. Urodynamic values were recorded continuously using data acquisition software (LABORIE, Canada). The following cystometric parameters were recorded and analyzed in this study: maximal micturition pressure at the start of micturition, micturition frequency, and inter-micturition interval.

### Statistical analysis

Data were represented as mean ± SEM of n strips in organ bath experiments and N mice in histological-examination/cystometry experiments. Significant differences were determined by Student's *t*-test. Only results with values of *p* < 0.05 were considered significant.
